# Yoghurt consumption is associated with changes in the composition of the human gut microbiome and metabolome

**DOI:** 10.1186/s12866-021-02364-2

**Published:** 2022-02-03

**Authors:** Caroline Ivanne Le Roy, Alexander Kurilshikov, Emily R. Leeming, Alessia Visconti, Ruth C. E. Bowyer, Cristina Menni, Mario Fachi, Hana Koutnikova, Patrick Veiga, Alexandra Zhernakova, Muriel Derrien, Tim D. Spector

**Affiliations:** 1 Department of Twin Research & Genetic Epidemiology, King’s College London, London, SE1 7EH UK; 2 Department of Genetics, University of Groningen, University Medical Center Groningen, Groningen, the Netherlands; 3 Danone Nutricia Research, Palaiseau, France

**Keywords:** Yoghurt, gut microbiome, 16S rRNA and whole shotgun metagenomic sequencing, diet, metabolomics, *Bifidobacterium animalis*, *Streptococcus thermophilus*, healthy eating

## Abstract

**Background:**

Yoghurt contains live bacteria that could contribute *via* modulation of the gut microbiota to its reported beneficial effects such as reduced body weight gain and lower incidence of type 2 diabetes. To date, the association between yoghurt consumption and the composition of the gut microbiota is underexplored. Here we used clinical variables, metabolomics, 16S rRNA and shotgun metagenomic sequencing data collected on over 1000 predominantly female UK twins to define the link between the gut microbiota and yoghurt-associated health benefits.

**Results:**

According to food frequency questionnaires (FFQ), 73% of subjects consumed yoghurt. Consumers presented a healthier diet pattern (healthy eating index: beta = 2.17 ± 0.34; *P* = 2.72x10^−10^) and improved metabolic health characterised by reduced visceral fat (beta = −28.18 ± 11.71 g; *P* = 0.01). According to 16S rRNA gene analyses and whole shotgun metagenomic sequencing approach consistent taxonomic variations were observed with yoghurt consumption. More specifically, we identified higher abundance of species used as yoghurt starters *Streptococcus thermophilus* (beta = 0.41 ± 0.051; *P* = 6.14x10^−12^) and sometimes added *Bifidobacterium animalis* subsp. *lactis* (beta = 0.30 ± 0.052; *P* = 1.49x10^−8^) in the gut of yoghurt consumers. Replication in 1103 volunteers from the LifeLines-DEEP cohort confirmed the increase of *S. thermophilus* among yoghurt consumers. Using food records collected the day prior to faecal sampling we showed than an increase in these two yoghurt bacteria could be transient. Metabolomics analysis revealed that *B. animalis* subsp. *lactis* was associated with 13 faecal metabolites including a 3-hydroxyoctanoic acid, known to be involved in the regulation of gut inflammation.

**Conclusions:**

Yoghurt consumption is associated with reduced visceral fat mass and changes in gut microbiome including transient increase of yoghurt-contained species (*i.e. S. thermophilus* and *B. lactis*).

**Supplementary Information:**

The online version contains supplementary material available at 10.1186/s12866-021-02364-2.

## Background

Yoghurt is widely consumed across the world, with the highest consumption registered in Europe [1]. Yoghurt is produced by fermenting milk with two lactic acid-producing bacteria, *Lactobacillus delbrueckii* subsp. *bulgaricus* (*L. bulgaricus*) and *Streptococcus thermophilus* (*S. thermophilus*) [2]. For most consumers, yoghurt generally falls under a wider umbrella of fermented milk products containing any *Lactobacillus/Lacticaseibacillus* (such as *L. casei*, *L. paracasei*, *L. rhamnosus*, *L. acidophilus*) or *Bifidobacterium* species (mostly *B animalis* subsp. *lactis B. lactis*)*.* Yoghurt consumption is associated with reduced body weight gain and incidence of type 2 diabetes in epidemiological studies [3–5]. Randomized controlled trials have shown that yoghurt intake reduces body fat in obese subjects and insulin resistance in obese women with non-alcoholic fatty liver disease (NAFLD) and metabolic syndrome (MetX) [6, 7].

Yoghurt contains on average 10^8^ colony-forming units (CFU)/g of live bacteria that can eventually incorporate the community of commensal microbes residing within the human gut [8]. The yoghurt bacteria, *S. thermophilus* and *L. bulgaricus* survive the gastrointestinal (GI) transit but generally reach low faecal concentrations (10^4^ to 10^6^ CFU/g faeces) in comparison with resident microbes [8–12]. Other strains contained in fermented milks such as *Bifidobacterium animalis* may better survive the transit and reach alive the colonic compartment with a higher abundance (up to 10^8^ CFU/ g faeces [13, 14]), indicating that they may have an increased contribution to microbiota changes compare to yoghurts starters. Due to the ability of bacteria to produce metabolites and to compete for substrates, introduction of a new species has the potential to modify the ecosystem structure [15]. Accordingly, in a 4-week intervention study on subjects with irritable bowel syndrome, the consumption of fermented milk was associated with increases of bacteria used in fermentation, including *B. lactis* [16]. In the same study, an increase in butyrate-producing metagenomic species was described as well as a decrease of the pathobiont *Bilophila wadsorthia* implying that fermented dairy product consumption may result in modifications of the composition but also the metabolic activity of the gut microbiota [16]. More recently in a 24-week intervention conducted in obese women with NAFLD and MetX, yoghurt consumption improved insulin resistance and changed the abundance of some members of *Firmicutes* compared to milk [6]. Thus, part of the beneficial effects of fermented milk product including yoghurt are thought to be mediated *via* modulation of the gut ecosystem.

Given high inter-subject gut microbiota variability, larger studies are required in order to fully characterise the role of fermented dairy product consumption in shaping this ecosystem [17]. Epidemiological studies have reported associations between yoghurt or fermented milk consumption and the composition of the gut microbiota. For instance, Zhernakova *et al*, found a positive association between the frequency of a specific fermented milk product consumption and gut microbiota diversity in a population of over 1000 subjects [18]. Furthermore, study of 260 volunteers revealed that those consuming yoghurt presented elevated levels of *S. thermophilus* in their gut while an increase in *Bifidobacterium* species was observed in *Bifidobacterium*-containing fermented milk consumers [19]. Finally, using a targeted approach Suzuki *et al* described that yoghurt and fermented dairy products consumption in 250 young Japanese adults was associated with increased levels of *Lactobacillus* and decreased *Staphylococcus* [20]. However, none of these studies conducted an in-depth untargeted analysis of the association between yoghurt consumption and the gut microbiome while considering all relevant confounders.

The aim of this study was to assess if yoghurt consumption is associated with changes of the gut microbiome and its metabolic activity and concomitant positive health outcomes in over 1000 aging twins from the TwinsUK cohort.

## Results

### Yoghurt consumption is associated with reduced visceral fat mass and healthier dietary habits

In total, 4117 volunteers from the TwinsUK cohort completed a Food Frequency Questionnaire (FFQ) between 1993 and 2015. This included 1092 volunteers who reported to never consume yoghurt and 3025 at least once a week (Table 1). The latter group could be split in 1900 low (1–5 times a week) and 1125 high (more than 5 times a week) consumers. The average yoghurt intake in the consumer group was of 4.67 times per week. The two groups were relatively homogeneous in terms of demographic characteristics apart from a significant enrichment in volunteers with a smoking history in the non-yoghurt consumer group (Fisher’s exact test *P* = 0.005). Yoghurt eaters presented on average lower visceral fat mass (VFM) (beta = −30.68 ± 11.73 g; *P* = 0.009) and reduced insulin levels (beta = −2.47 ± 1.15 pmol/L; *P* = 0.03) after correction for age, gender, body mass index (BMI) and family structure. Using the Healthy Eating Index (HEI), calculated as described by Bowyer et al., without yoghurt variables (Table 1) we observed that yoghurt consumption was associated with a healthier diet pattern (beta = 2.17 ± 0.34; *P* = 2.72 x10^−10^). Subsequent analysis of the 12 components used to calculate the HEI revealed that this could partly be explained by a significant increase in fruit, grain and dairy consumption and a decrease in protein intake in the yoghurt consumer group (Table 1). Consequently, the HEI was integrated as a confounding factor besides the aforementioned covariates in subsequent analyses. In fact, whilst the previously reported association between VFM and yoghurt consumption remained significant after inclusion of HEI in our model (VFM; beta = −28.18 ± 11.72 g; *P* = 0.016) only a trend was maintained for the association with insulin (beta = −2.03 ± 1.14 pmol/L; *P* = 0.076).

**Table 1:** Population characteristics, divided between yoghurt consumers and non-consumers. *P* values were generated using linear mixed effect models where yoghurt consumption was used a predictor and age, sex and BMI were included as fixed effects and family structure as a random effect (M1), M2 was calculated by adding HEI as covariate to M1. * *P*-value obtained from Fisher’s exact test to evaluate enrichment of a given phenotype (smoking or type 2 diabetes) in the yoghurt consumer group

	Yoghurt consumers	Yoghurt non-consumers	*P*-value M1	*P*-value M2
	(*n* = 3025)	(*n* = 1092)		
Yoghurt intake (1–5 times a week)	1900	0		
Yoghurt intake (> 5 times a week)	1125	0		
Total yoghurt intake (times/week)	4.67 ± 3.08	0.03 ± 0.08		
Age (year)	67.6 ± 12.6	65.8 ± 14.3		
Sex (% Female)	0.937	0.857		
Smoking history (%)	45.73%	50.70%	0.005*	
***Metabolic parameters***				
T2DM (%)	6.99%	9.50%	0.12*	
BMI (kg/m^2^)	25.43 ± 0.08	25.22 ± 0.13	0.402	0.292
Body weight (kg)	67.45 ± 0.24	67.32 ± 0.39	0.211	0.155
Body fat mass (%)	47.27 ± 0.62	49.09 ± 1.08	0.547	0.679
Visceral fat mass (g)	539.51 ± 6.60	573.72 ± 11.46	0.009	0.016
Fasting glucose (mmol/L)	4.83 ± 0.01	4.83 ± 0.02	0.307	0.333
Fasting insulin (pmol/L)	39.57 ± 0.16	38.73 ± 0.28	0.031	0.076
C-reactive protein (mg/l)	1.85 ± 0.05	1.96 ± 0.08	0.157	0.311
Alanine aminotransferase (Ul/L)	20.83 ± 0.18	20.99 ± 0.28	0.433	0.39
***Dietary habits*** *(points)*				
HEI	59.97 ± 9.03	57.22 ± 11.08	2.72x10^−10^	
Whole fruit	4.93 ± 0.46	4.64 ± 1.03	3.72 x10^−30^	
Total fruit	4.89 ± 0.51	4.49 ± 1.09	9.75 x10^−46^	
Whole grains	8.16 ± 2.64	6.74 ± 3.41	2.7 x10^−35^	
Dairy	5.58 ± 2.34	5.30 ± 2.7	0.002	
Total protein	2.23 ± 0.65	2.35 ± 0.78	4.58 x10^−09^	
Sea plant protein	4.30 ± 0.94	4.39 ± 0.99	0.001	
Greens and beans	4.52 ± 0.94	4.53 ± 0.99	0.907	
Total vegetable	3.75 ± 1.22	3.77 ± 1.29	0.269	
Fatty acids	3.76 ± 2.40	3.62 ± 2.73	0.467	
Refined grains	1.10 ± 2.65	1.05 ± 2.73	0.592	
Sodium	7.84 ± 2.05	8.02 ± 2.25	0.061	
Empty calories	8.86 ± 4.82	8.24 ± 5.63	0.072	
***Other datasets***				
16S rRNA gene sequencing (n)	1057	400		
Shotgun metagenomic sequencing (n)	400	144		
Faecal metabolomic (n)	309	110		

### Yoghurt consumption is associated with changes in the composition of the gut microbiome

**Fig. 1:**
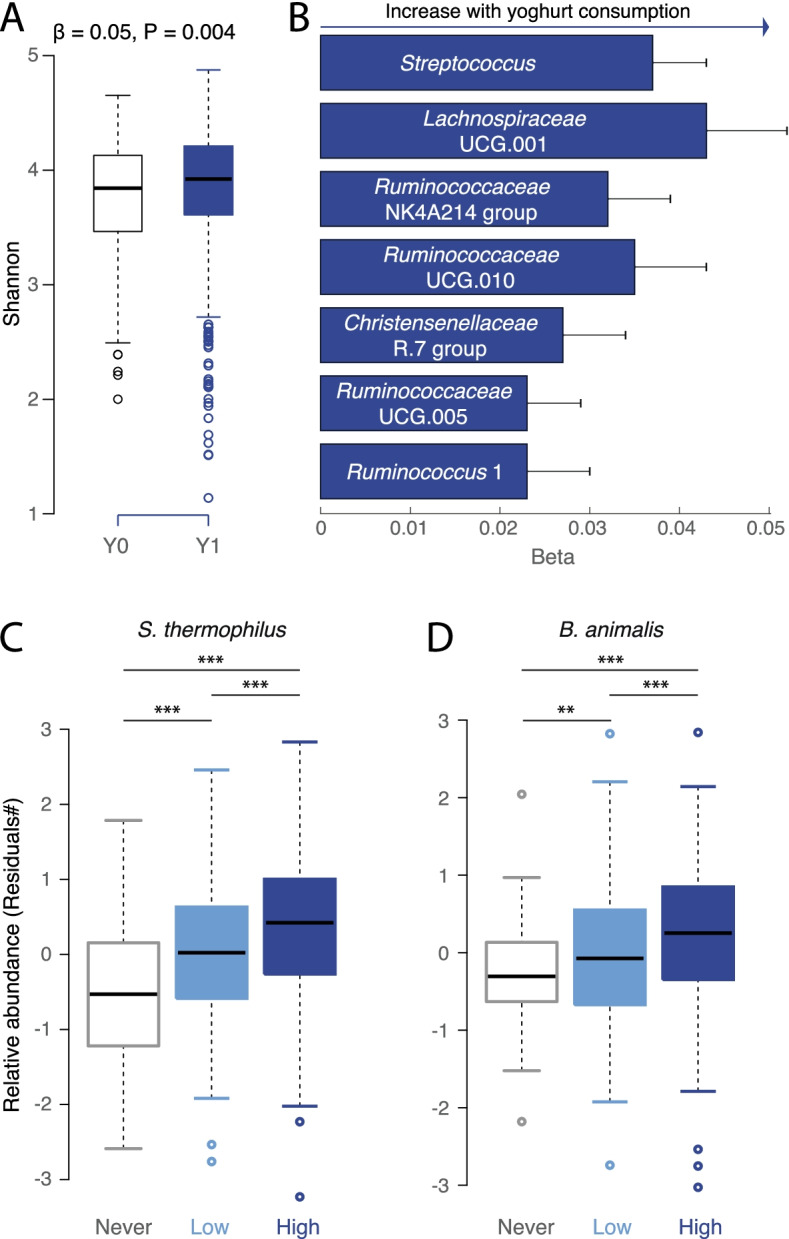
Yoghurt consumption is associated with a distinct gut microbiome signature. **A** Boxplot representing the association between yoghurt consumption and gut microbiota alpha diversity for 16S rRNA gene dataset. **B** Effect size of the significant (Bonferroni threshold) association between yoghurt intake and seven genera. **C** and **D**. Boxplot comparing residuals of *S. thermophilus* (C.) and *B. animalis (D.) *between non-yoghurt consumers (never, white, *n* = 144) and low (light blue, *n* = 183) or high (dark blue, *n* = 217) yoghurt consumers; ** *p* < 0.01; *p* < 0.001 according to linear regression results (‘*lme4’* package in R) including family structure as random effect and age, BMI, HEI and sex as fixed effects

Gut microbiota was analysed in a subset of the population using whole shotgun metagenomic sequencing (400 yoghurt eaters and 144 yoghurt non-eaters) and 16S rRNA sequencing (1057 yoghurt eaters and 400 yoghurt non-eaters, that overlap with the shotgun metagenomic data). While yoghurt consumption was associated with a higher alpha diversity according to the 16S rRNA sequencing data after correction for age, BMI, sex, HEI and family structure (Fig. 1A & Supplementary Table 2; Shannon: beta = 0.05 ± 0.02; *P* = 0.004), significance was not reached for shotgun metagenomics (Shannon: beta = − 0.01 ± 0.07; *P* = 0.87). In both datasets, yoghurt consumption was not associated with variations in beta diversity. Additionally, genus-level analyses, based on 16S rRNA gene sequencing, found that the relative abundance of seven genera were significantly increased in the yoghurt consumer group, including *Streptococcus*, and unidentified genera within *Lachnospiraceae* (UCG001), *Christensenellaceae* (R7) and *Ruminococcaceae* (Fig. 1B & Supplementary Table 3).

Whole shotgun metagenomic sequencing analysis revealed that two species out of the 541 identified in the population were significantly positively associated with yoghurt consumption (Supplementary Table 4), namely *S. thermophilus* (beta = 0.41 ± 0.05 and *P* = 6.14x10^−12^) and *B. animalis* (beta = 0.30 ± 0.05 and *P* = 1.49x10^−8^). We also observed a dose dependent response for these two species (Fig. 1C&D) as high yoghurt consumers presented greater levels of *B. animalis* and *S. thermophilus* than low consumers. A targeted analysis suggests that *B. animalis* subsp. *lactis* (beta = 0.36 ± 0.07; *P* = 7.89x10^−7^) rather than *B. animalis* subsp. *animalis* (*P* = 0.25) was associated with yoghurt consumption (Supplementary Fig. 1 B&C). Thus, *S. thermophilus and B. animalis* subsp. *lactis* are markers of consumption of a fermented milk containing this species. Finally, at the functional level, only the peptidoglycan biosynthesis IV pathway was enriched in the gut of yoghurt consumers (beta = 0.31 ± 0.052, *P* = 6.99x10^−7^).

We sought to replicate our analyses in 1103 volunteers from the LifeLines-DEEP cohort and used the dairy fermented product consumption as a proxy for yoghurt consumption using linear regression accounting for gender, age and BMI but not diet (HEI not available). Higher proportion of yoghurt consumers (91%) was reported in LifeLines-DEEP cohort compared to that of TwinsUK (64%). 1010 volunteers were identified as consumers and 93 as non-consumers based on FFQ (Cohort description Supplementary Table 2). We found that *S. thermophilus* (0.008 ± 0.001; *P* = 4.77x10^−16^) but not *B. animalis* (*P* = 0.38) was associated with yoghurt consumption. Unlike that observed in the TwinsUK cohort, alpha diversity metrics were not associated with yoghurt intake according to 16S rRNA and whole shotgun metagenomic sequencing data (Shannon 16S rRNA: *P* = 0.64; Shannon shotgun metagenomics: *P* = 0.21).

### The increase of yoghurt-bacteria in the gut is transient

**Fig. 2:**
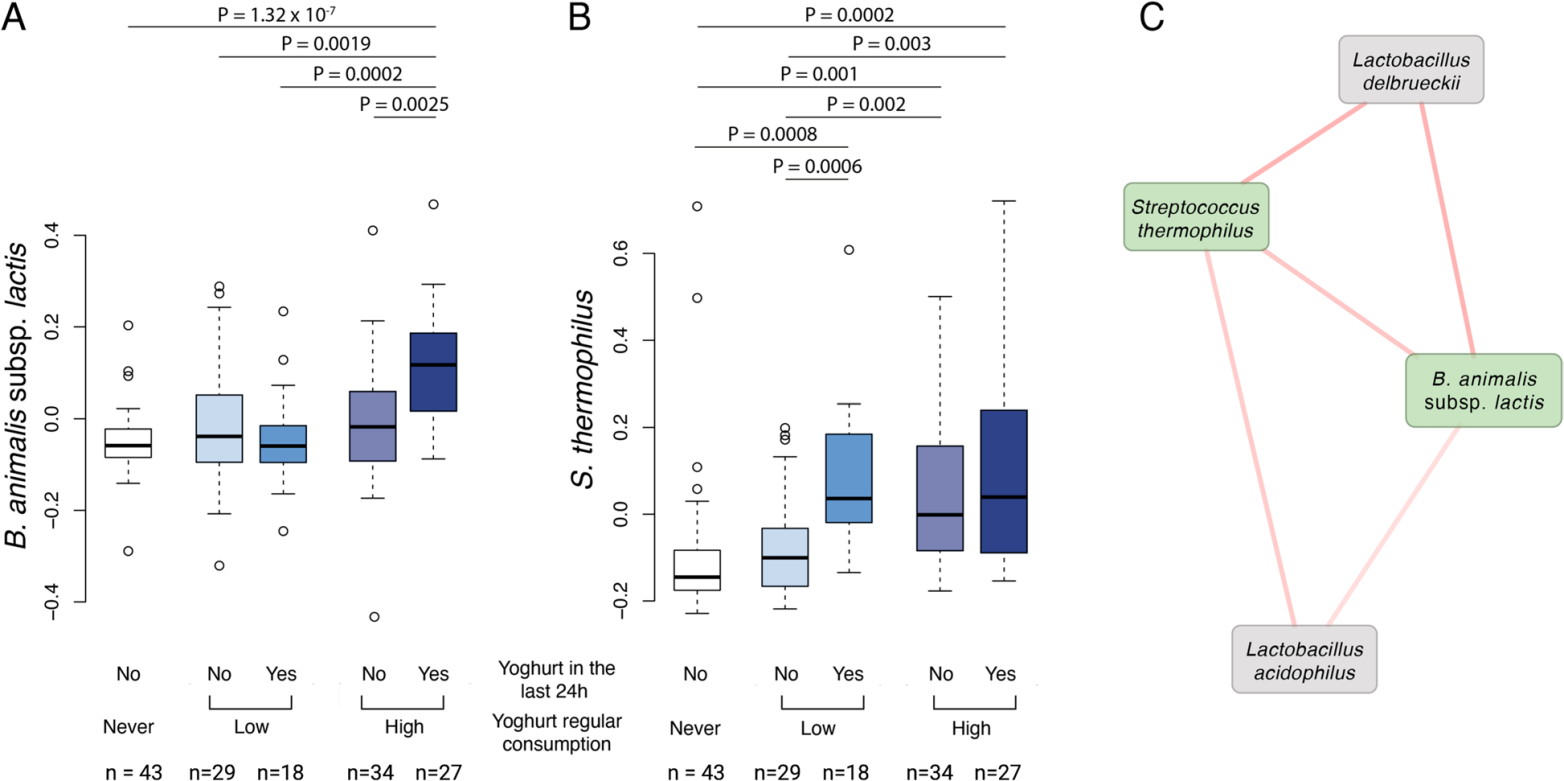
*S. thermophilus* and *B. animalis* subsp. *lactis* increase momentarily in the gut following yoghurt consumption. **A** Residuals of the relative abundance of *B. animalis* subsp. *lactis* after correction for age, gender, BMI and HEI, according to yoghurt eating habits and consumption the day prior to faecal sample collection. *P* values were obtained from linear regression including family structure as random effect and age, BMI, HEI and sex as fixed effects. **A** Residuals of the relative abundance of *S. thermophilus* after correction for age, gender, BMI and HEI, according to yoghurt eating habits and consumption the day prior to faecal sample collection. **C** ‘Yoghurt’ sub-network in which *S. thermophilus* and *B. animalis* subsp. *lactis* are included (green boxes). Red lines represent positive associations between two species and their thickness the strength of this association

To evaluate the temporal length of the increase in the two bacteria associated with yoghurt consumption we used the same data in a subset of 151 volunteers who reported consuming yoghurt on the day prior providing the faecal sample *via* food records. Out of the 151 participants, 46 had been classified as non-yoghurt consumers according to the FFQs and none of them reported to consume yoghurt according to the food records. However, in the yoghurt consumer group (according to FFQs), only 44% of high consumers also reported yoghurt consumption in their food records against 38% in the low consumption group. This suggests that participants of the high consumption group may have over reported yoghurt consumption in the FFQs. This enabled to question whether variations in *S. thermophilus* or *B. animalis* subsp. *lactis* is conditioned by yoghurt consumption within the last 24 h. An increase in *B. animalis* subsp. *lactis* was only observed in people who eat yoghurt the day before sampling and were classified as high consumers (Fig. 2A). On the other hand, significant increases in *S. thermophilus* were observed in volunteers who consumed yoghurt the day prior to sampling regardless of their frequency of consumption (Fig. 2B). Besides high yoghurt consumers who had not eaten yoghurt the day before sampling also had elevated levels of *S. thermophilus* (Fig. 2B). Finally, there was no correlation between reported quantity of yoghurt intake in the food records data and the relative abundance of the two bacteria (*n* = 45 volunteer not eating yoghurt excluded, *B. animalis* subsp. *lactis*: Spearman’s rho = − 0.01 and *P* = 0.91; *S. thermophilus*: Spearman’s rho = 0.09 and *P* = 0.55).

Using a co-occurrence network approach based on Spearman’s correlations in the full dataset (*n* = 544), we next observed that *B. animalis* subsp. *lactis* and *S. thermophilus* were found to co-occur with other lactic acid bacteria (*Lactobacillus delbrueckii* and *Lactobacillus acidophilus*) (Fig. 2C) but none of the other commensal species. Together, these results suggest that the passage of *S. thermophilus* and *B. animalis* subsp. *lactis* through the GI tract may be considered as transient.

### Association between yoghurt bacteria and other parameters

Finally, we aimed to evaluate the effect of yoghurt consumption on faecal metabolic composition. To this end, we compared the faecal metabolome of yoghurt consumers (*n* = 309) versus non-consumers (*n* = 110) using linear mixed effect models and adjusted for age, gender, BMI, HEI and family structure. In total 1116 metabolites were measured, including 850 of known chemical identity. Out of these, only one metabolite, 5alpha-androstan-3beta,17beta-diol disulfate, a steroid was found to be significantly decreased in the stools of yoghurt consumers (beta = −0.35 ± 0.07; *P* = 1.56x10^−9^; Supplementary Table 6).

**Fig. 3:**
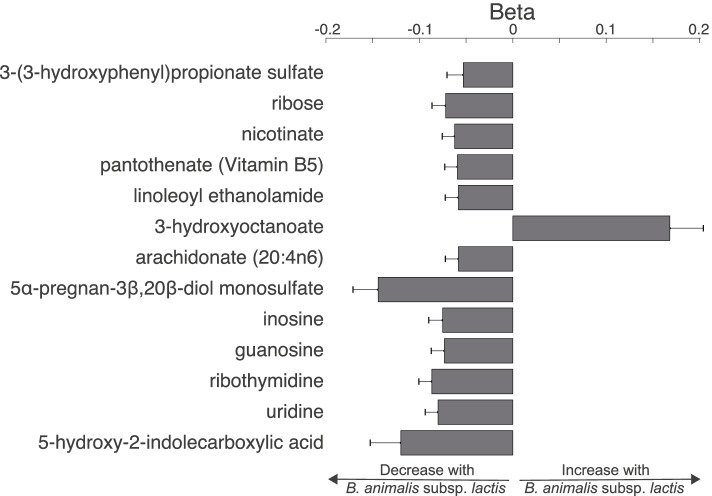
*B. animalis* subsp. *lactis* is associated with faecal metabolites. Beta of the significant associations (*P* = 0.05/*N* = 850) between faecal metabolites and *B. animalis* subsp. *lactis* calculated using a linear mixed effect model correcting for age, sex, BMI, HEI and family structure

We next explored the faecal metabolic footprints of *B. animalis* subsp. *lactis* and *S. thermophilus* by looking at their association with the 850 known faecal metabolites measured in a subset of 340 volunteers from whom both datasets (metagenomic and metabolomic) were available. While *S. thermophilus* was only associated with anacardic acid (beta = 0.36 ± 0.041 and *P* = 9.62x10^−17^), *B. animalis* subsp. *lactis* was significantly associated with 13 faecal metabolites (Fig. 3, Supplementary Table 7). Notably a positive association was observed between *B. animalis* subsp. *lactis* and 3-hydroxyoctanoic acid, an agonist of the hydroxycarboxylic acid receptor 3 (HCA_3_) whereas all other associations were negative.

Last, we tested if *S. thermophilus* or *B. animalis* subsp. *lactis* that are increased in the gut of yoghurt consumers, were also associated with VFM and fasting insulin that were both associated with yoghurt intake. However, none of the results were significant (Supplementary Table 5).

## Discussion

We conducted an in-depth, large population-based analysis of the effect of yoghurt consumption on the gut microbiome characterised *via* 16S rRNA and whole shotgun metagenomic sequencing while accounting for covariates, such as age, gender, BMI and most importantly habitual diet. Analysis of 16S rRNA gene sequencing data but not whole shotgun metagenomic sequencing on a lower sample size, revealed that gut microbiota from yoghurt consumers harboured a higher alpha-diversity than that of non-consumers. In an independent cohort (LifeLines-DEEP), shotgun sequenced data and 16S rRNA, alpha diversity was not increased in the gut of yoghurt consumers, which may be due to the fact that only a low proportion of the population (8.4%) could be clarified as non-yoghurt consumers, limiting statistical power. Taken together, our results suggest that larger sample size and comparable group size is needed to elucidate the contribution of yoghurt consumption to gut microbiota alpha diversity, obscured by technical and population variability.

Yoghurt consumption was associated with an increase in *Streptococcus* but also *Christensenella* and *Ruminococcaceae*. The observed increase in *Ruminococcus* genus in our study is in contradiction with previously published results where a 24-week yoghurt intervention was followed by a decrease in *Ruminococcus* [6]. This discrepancy may be due to the differences in population and statistical analysis. We observed an increase in *Streptococcus* with 16S RNA gene sequencing that was further assigned to *S. thermophilus* by Shotgun sequencing, also observed in LifeLines-DEEP cohort. Besides, *B. animalis* subsp. *lactis* added to yoghurt products, was found to be increased in the yoghurt consumer group of the TwinsUK cohort. Both bacterial species/subspecies are used in the making of fermented milk products and were found to be increased in the gut of yoghurt consumers in several other observational studies [19, 20]. Yet, the *B. animalis* subsp. *lactis* observation was not replicated in the LifeLines-DEEP cohort, potentially indicating that dairy products consumed by participants of in the LifeLines-DEEP cohort were less enriched in *B. animalis* subsp. *lactis.* Since *B. animalis* subsp. *lactis* is a species adapted to the GI tract and known to reach the colon alive, one can make the hypothesis that the higher alpha diversity observed in the TwinsUK is specific to the consumption of *B. animalis* subsp. *lactis* enriched products, less consumed in the the LifeLines-DEEP cohort. Thus, following yoghurt consumption, gut microbiome composition is characterised by an increase of the bacteria ingested from the product, which also implies that a fine description of the bacteria composition of yoghurt is needed to further define their effects on the gut microbiome.

Using a combination of FFQs and food record data we observed that the increase of these two bacteria in the human gut following yoghurt consumption might be transient as highly determined by consumption of yoghurt within the 24 h preceding the faecal sample. Transient detection of yoghurt / fermented milk strains has been shown in various clinical studies [13, 21]. While *S. thermophilus* and *B.animalis* subsp. *lactis* are able to thrive in simple communities, they are outcompeted by resident bacteria in more complex ecosystems through colonisation resistance. Our data confirm that yoghurt-bacteria are transient members of the gut microbiome and do not durably engraft within the gut lumen. Interestingly, a recent study showed that *Streptococcus thermophilus* was one of the most prevalent lactic acid bacteria detected in 9445 metagenomes from human samples [22].

Co-occurrence network analysis indicated that *B. animalis* subsp. *lactis* and *S. thermophilus* belonged to a subnetwork composed exclusively of lactic acid bacteria, with a co-variance most likely explained by yoghurt consumption. This may relate to heterogeneity in the permissiveness of the resident ecosystem to integrate ingested strain [23–27]. It is not possible to exclude that both bacteria, *B. animalis* subsp. *lactis* and *S. thermophilus*, may exert a greater impact on the microbial ecosystem in the small intestine where ingested strains outnumber resident microbes [17]. Alternatively, yoghurt bacteria might exert their effect locally as they can adhere to some extend to intestinal cells [12], which would need to be ascertained using more invasive technics [23]. Taken together, our study shows that while cross-sectional cohort can reveal association between transient microbes and gut resident gut microbiome, longitudinal settings coupled with population stratification and alternatively biogeographical sampling of the gut microbiome are warranted to better decipher the detailed nature of these interactions. Besides, these results infer that the design of fermented dairy product may in the future benefit from the addition of strains capable of integrating and / or covarying with the gut ecosystem more efficiently in the context of precision medicine [28].

We observed that *B. animalis* subsp. *lactis* was associated with 13 faecal metabolites. Among these, we reported a positive association with 3-hydroxyoctanoic acid, an agonist of the hydroxycarboxylic acid receptor 3 (HCA_3_) that was not reflected in blood metabolome. HCA_3_ is expressed in enterocytes and its inactivation by 3-hydroxyoctanoic acid mediates a reduction of inflammation [29]. Our results are in line with a recent study demonstrating that metabolites of lactic acid bacteria present in fermented foods, *i.e.* 3-hydroxyoctanoic acid can inactivate HCA_3_ [30]. In accordance with the literature, we reported that yoghurt consumption was associated with markers of metabolic health, namely a decrease in VFM and fasting insulin. Even though these two parameters have previously been linked with gut microbiome alpha-diversity and the abundance of some genera such as *Christensenella*, that were both increased in the gut of yoghurt consumers [31–33], neither of the two yoghurt bacterial species were linked to these phenotypes. This implies that factors others than the increase in *S. thermophilus* and *B. animalis* subsp. *lactis* may also be at play. Yet when exploring the effects of yoghurt consumption of faecal metabolome, only a significant decrease in 5alpha-androstan-3beta,17beta-diol disulfate was observed in the yoghurt consuming group. This metabolite is an endogenous steroid that is mostly known for its potential role on the hypothalamo-pituitary–adrenal axis [34] that is involved in stress response [35].

As previously described in epidemiological studies [36–40], we reported that yoghurt consumption was linked with healthy dietary habits. In the present study we intended to account for this bias by adjusting for habitual diet which in part attenuated the original results. However, the HEI used here as a covariate may not capture in full diet quality and specifically, the individual contribution of fruit, whole grain and protein that were significantly different between yoghurt consumers and non-consumers. The HEI was generated based on FFQs that may be biased by the fact that volunteers tend to over report healthy foods in self-reported questionnaires [41]. The latter may also have impacted the assessment of yoghurt consumption. Besides, answers to these FFQs provided limited information regarding the type of yoghurt consumed while it had been demonstrated that distinct yoghurt types (natural vs. sweetened) may impact differently the gut microbiome [42]. It is therefore necessary to highlight that the results presented in this manuscript solely report the global effects of yoghurt consumption without investigating in depth the potential effects of individual product types such as sugar content or ferment use. Finally, we reported that FFQs were not always collected at the same time as the biological samples used in this study. Although responses to FFQs that capture habitual diet tend to be stable overtime [43], it is not possible to exclude that some participants may have changed their dietary habits during this time span therefore affecting the accuracy of the results. Further, one of the main limitations of the present work is its inherent observational nature that does not allow inference of causal relationships. Finally, associations reported here were observed in a predominantly older white female British cohort and may not apply to other populations. Nevertheless, some of the microbiome results were replicated in an independent Dutch cohort and generally reflected current literature suggesting observations reflect wider patterns applicable to other populations.

## Conclusions

Yoghurt consumption is associated with a healthier dietary pattern, reduced visceral fat mass and a transient increase in the gut of bacterial species used in the making of yoghurt, namely *S. thermophilus* and *B. animalis* subsp. *lactis*. *S. thermophilus* increase was replicated in an independent European cohort whereas the positive association between *B. animalis* subsp. *lactis* and yoghurt consumption was cohort dependent. Finally, *B. animalis* subsp. *lactis* appeared to be significantly associated with a number of faecal metabolites including 3-hydroxyoctanoic acid that could be involved in yoghurt-associated health benefits.

## Methods

### Study population

The analysis included individuals, enrolled on the TwinsUK registry, a registry of extensively phenotyped, mainly female, adult monozygotic (MZ) and dizygotic (DZ) twins from the UK [44]. Twins were recruited nation-wide primarily through media campaigns. Ethical approval has been obtained from St. Thomas’ Hospital Research Ethics Committee and all subjects have undergone informed consent. This analysis included female and male twins, aged 18 to 89 years, who had completed at least one FFQ between 1994 and 2001, in 2007, and in 2014 and 2015. Faecal samples were collected between 2010 and 2015.

### Dietary assessment

Twins completed a 131-item FFQ that was developed and validated against pre-established nutrient biomarkers for the European Prospective Investigation into Diet and Cancer (EPIC) Norfolk [45, 46]. Processing of the FFQ, including subject exclusion and determination of nutrient intakes was completed by nutritionists from the University of East Anglia. Intake frequency of an average serving of listed foods was determined from a 9-point scale ranging from “Never or less than once/month” to “6+ per day”. The questionnaire was intended to capture average intakes in the past year. Nutrient intakes were determined via consultation with an established nutrient database [47]. Submitted FFQs were excluded if more than 10 food items were left unanswered, or if the total energy intake estimate derived from FFQ as a ratio of the subject’s estimated basal metabolic rate (determined by the Harris-Benedict equation [48]) was larger than two standard deviations outside the sample mean of this ratio (*i.e.*, < 0.52 or > 2.58).

### Yoghurt consumption

Two items included in the FFQ were used to determine yoghurt consumption: “Low fat yoghurt, fromage frais (125g carton)” and “Full fat or Greek yoghurt (125g carton)” which.

were merged into one variable. Twins reporting “Never or less than once/month” were considered non-consumers, twins reporting frequencies of “once a week” to “2-3 per day” were considered consumers. Twins reporting “1-3 per month” or from “4-5 per day” to “6+ per day” were not included.

### Healthy eating index (HEI)

The HEI (described as the best measure to capture the effect of diet on the gut microbiota) [49] was constructed as described by Guenther et al., [50] using dietary intakes estimated *via* the food frequency questionnaire (FFQ excluding “yoghurt” variables. The 13 components calculated based on the 131 FFQs entry used to construct the HEI were also used to identify association between yoghurt consumption and eating patterns.

### Health biomarkers

Health biomarkers were collected during clinical visits. Weight and height were measured at each visit and used to calculate body mass index (BMI). Body composition measurements such as visceral fat mass (VFM) and percentage of body fat were measured by total body dual- energy X-ray absorptiometry (DXA) whole-body scans following manufacturer’s recommendations (QDR Discovery W system; Hologic, Inc., Bedford, MA). Participants were asked to lie flat and straight during the DXA procedure for the full body scan, as previously reported [51]. Visceral fat mass was calculated from one cross section of the whole body at L4–L5 spinal segment (the two lowest vertebra of the lumbar spine), the typical location of a computed tomography (CT) slice and is estimated in grams. All scan printouts were reviewed by an expert reader to ensure proper positioning and analysis. Levels of blood biomarkers (insulin, glucose and C-reactive protein) were measured on blood samples collected upon arrival at the clinic (fasted).

### Faecal samples collection

Faecal samples were collected by the twins at home between 2010 and 2015 using the TwinsUK sample collection kit (mainly dry sarstedt tube). Following collection, samples were stored in the refrigerator for 2 days or less prior to their annual clinical visit at St. Thomas’ Hospital. Once the samples arrived with the clinical team, they were stored at −80 °C until further processing. The average time between faecal samples and FFQ completion was −0.81 years (SD: 1.77 years; range: ±5 years).

### 16S rRNA sequencing data

The composition of the gut microbiome was determined by 16S rRNA gene sequencing carried out at Cornell as previously described [52]. Following quality control (QC), amplicon sequence variances (ASVs) were generated using the DADA2 pipeline [53]. This technique presents the advantage of resolving differences down to single nucleotide level [54]. Generated ASVs were aggregated at different taxonomic levels before analysis. Shannon diversity metrics were generated as previously described [55].

### Shotgun metagenomics

Details of DNA extraction, library preparation, and sequencing are described elsewhere [Visconti et al., 2019]. Briefly, Nextera XT libraries were prepared manually following the manufacturer’s protocol (Illumina, PN. 15,031,942) and sequencing was performed on an Illumina HiSeq 2500 using SBS kit V4 Chemistry, with a read length of 2 × 125 bp. Sequencing of 1054 samples yielded an average number of reads of 54 million per sample before QC. Paired-end reads were processed using the YAMP pipeline [56]. In the QC step, identical reads, adapters, known artefacts, phix174 were removed, and then reads were quality trimmed (PhRED quality score < 10), and reads that became too short after trimming (N < 60 bp) were discarded. Singleton reads (*i.e.,*reads whose mate has been discarded) were retained. Finally, contaminant reads belonging to the host genome were removed. We removed 4 samples with <15 millions reads after QC, 37 with ecologically abnormal samples and 9 individuals not of European ancestry, resulting in 1004 samples with an average number of reads of 39 million, as previously described [57]. Next, YAMP was used to characterise the microbial community (*via* MetaPhlAn2, v. 2.6.0 [58];) and the microbial metabolic pathway they contribute to (via the HUMAnN2 pipeline, v 0.10.0; UniRef90 proteomic database [59];). This dataset consisted of 144 non-consumers and 400 consumers (183 had low yoghurt consumption and 217 high yoghurt consumption).

### Subspecies and Strain level analysis

Subspecies and strain annotation was performed using the quality-controlled whole-shotgun metagenomic sequencing data for 1004 individuals and BBsplit, a tool belonging to the BBMap suite (https://github.com/BioInfoTools/BBMap/blob/master/sh/bbsplit.sh) that bins reads by mapping to multiple references simultaneously, using a 99% similarity threshold. Ambiguous reads (*i.e.,* reads that map to several strains) were removed from the analysis. Fasta files for references genomes were downloaded from Progenomes. Statistical analysis was performed on zero-inflated log_10_ transformed relative abundances calculated based on the above annotation.

### Metabolic profiling

Metabolite ratios were measured from faecal samples by Metabolon, Inc., Morrisville, North Carolina, USA, by using an untargeted UPLC-MS/MS platform as previously described [60]. A total of 1116 metabolites were measured in 480 faecal samples with whole shotgun metagenomic data available, including 850 of known chemical identity used in this study. Metabolites were scaled by run-day medians, and log-transformed. Faecal metabolites were further scaled to have mean zero and standard deviation one. Metabolites that were indicated as below detection level (zero) were considered as not available (NA).

### Statistical analysis

We performed pairwise associations (‘*lme4’* package in R, version 3.6.1) between microbial measurements (alpha diversity; species and metabolic pathways relative abundances) and yoghurt consumption (consumer *vs.* non-consumer) using family structure as random effect, while age, BMI, sex, HEI as fixed effects. For 16S rRNA amplicon sequencing data, sequencing run, sequencing depth, who extracted the DNA, who loaded the DNA and sample collection method were added to the model as technical co-variates. Results were considered significant when passing a Bonferroni-derived threshold of *P* < 0.05/number of tests of each microbial dataset. The same approach was used to evaluate the association between yoghurt consumption and HEI and its components, where family structure, age, BMI and sex were included as covariates. To determine the percentage of inter-individual microbiome variance explained by yoghurt consumption (beta diversity) we performed Permutational Multivariate Analysis of Variance (PERMANOVA, ‘*Vegan’* package in R, version 3.6.1) on Bray-Curtis distances with 1000 permutations.

### Network visualisation

Networks were created in Cytoscape (https://cytoscape.org[61]) based on Spearman’s correlations between all species plus the two *B. animalis* subspecies (calculated using the all dataset with whole-shotgun metagenomic sequencing, *n* = 1004) that displayed a *p*-value passing a Bonferroni-derived threshold of *P* < 2.5x10^−7^.

### Estimated food record

Estimated food record *via* a written diary were collected from 151 participants from the TwinsUK cohort detailing 24-h of food and drink intake. All these participants provided 24-h records the day prior to faecal sample collection and also responded to a FFQ. Participants’ intakes were electronically processed by Abacus Ltd. using Dietplan software to calculate nutrition information and food portions in grams. A binary variable was applied to participants who consumed yoghurt *vs.* those who did not consume yoghurt (cut-off of at least one yoghurt reported in the record). The quantity of yoghurts consumed by a participant was determined by the (unweighted) quantity of yoghurt entries within the 24 h period.

### Replication in the LifeLines-DEEP cohort

Replication of significant findings was pursued in the LifeLines-DEEP cohort where 1010 yoghurt consumers were compared to 93 non-yoghurt consumers as reported through FFQs. 16S rRNA and whole shotgun metagenomics sequencing data were processed as previously described by Zhernakova et al. [18]. Association between yoghurt consumption and microbiome variables of interest were tested using linear regression adjusting for age, sex and BMI. Results displaying a *p* value below Bonferroni threshold (0.05/number of tests) were considered significant.

## Supplementary Information

**Additional file 1: Supplementary Figure 1.** Association between yoghurt consumption and *B. animalis* subsp. *lactis* . A. Scatter plot of the correlation between *B. animalis* subsp. *lactis* and *B. animalis* subsp. *animalis*. B. Boxplot picturing the association between frequency of yoghurt consumption and *B. animalis* subsp. lactis. C. Boxplot picturing the association between frequency of yoghurt consumption and *B. animalis* subsp. *animalis*. Results were obtained from linear regression (lme4 package in R) including family structure as random effect and age, BMI and sex as fixed effects.

**Additional file 2: Supplementary Table 1.** Description of the LifeLines-DEEP cohort.

**Additional file 3: Supplementary Table 2.** Association between yoghurt consumption gut microbiota alpha diversity. Results were obtained by fitting linear mixed effect model where alpha diversity metrics generated from 16S rRNA and shotgun metagenomics data from two cohort were used as a response to level of yoghurt consumption and BMI, sex and age, as well as HEI and family structure for TwinsUK only, were used as covariates.

**Additional file 4: Supplementary Table 3.** Association between yoghurt consumption and taxa (16S rRNA sequencing). Results were obtained by fitting linear mixed effect model where taxa were used as a response to level of yoghurt consumption and BMI, sex, age, HEI and family structure were used as covariates.

**Additional file 5: Supplementary Table 4.** Association between yoghurt consumption and gut microbiome species (shotgun metagenomics). Results were obtained by fitting linear mixed effect model where species were used as a response to level of yoghurt consumption and BMI, sex, age, HEI and family structure were used as covariates.

**Additional file 6: Supplementary Table 5.** Association between dairy fermented bacterial species (*B. animalis* subsp. *lactis* and *S. thermophilus*) and blood and phenotypical parameters associated with yoghurt consumption. Results were obtained by fitting linear mixed effect model where phenotypes and blood parameters were used as a response to species levels and BMI, sex, age, HEI and family structure were used as covariates.

**Additional file 7: Supplementary Table 6.** Association between yoghurt consumption and faecal metabolites. Results were obtained by fitting linear mixed effect model where metabolites were used as a response to level of yoghurt consumption and BMI, gender, age, HEI and family structure were used as covariates.

**Additional file 8: Supplementary Table 7.** Association between *B. animalis* subsp. *lactis* and faecal metabolites. Results were obtained by fitting linear mixed effect model where metabolites were used as a response to level of yoghurt consumption and BMI, gender, age, HEI and family structure were used as covariates. Only significant results (passing Bonferroni threshold *P* < 5.88x10^−5^).

## Data Availability

The raw metagenomic sequences are available from the European Nucleotide Archive website (study accession number: PRJEB32731). TwinsUK 16S rRNA gene sequencing data are available from the BioProject database under accession code PRJEB13747. All other phenotypical information’s may be available upon request to the department of Twin Research at King’s College London (http://www.twinsuk.ac.uk/data-access/accessmanagement/).

## Abbreviations

ASV: Amplicon sequence variances
BMI: Body mass index
bp: Base pair
CFU: Colony-forming units
CT: Computed tomography
DXA: Dual- energy X-ray absorptiometry
DZ: Dizygotic
EPIC: European Prospective Investigation into Diet and Cancer
FFQ: Food frequency questionnaires
GI: Gastrointestinal
HEI: Healthy eating index
MetX: Metabolic syndrome
MZ: Monozygotic
NAFLD: Non-alcoholic fatty liver disease
PERMANOVA: Permutational Multivaraite Anlaysis of Variance
QC: Quality control
SD: Standard deviation
subsp.: Subspecies
UK: United kingdome
UPLC-MS/MS: Ultra performance liquid chromatography - tandem mass spectrometer
VFM: Visceral fat mass
