# Correction to: Yoghurt consumption is associated with changes in the composition of the human gut microbiome and metabolome

**DOI:** 10.1186/s12866-022-02482-5

**Published:** 2022-02-28

**Authors:** Caroline Ivanne Le Roy, Alexander Kurilshikov, Emily R. Leeming, Alessia Visconti, Ruth C. E. Bowyer, Cristina Menni, Mario Fachi, Hana Koutnikova, Patrick Veiga, Alexandra Zhernakova, Muriel Derrien, Tim D. Spector

**Affiliations:** 1 Department of Twin Research & Genetic Epidemiology, King’s College London, London, SE1 7EH UK; 2 Department of Genetics, University of Groningen, University Medical Center Groningen, Groningen, the Netherlands; 3 Danone Nutricia Research, Palaiseau, France

**Correction to: BMC Microbiol 22, 39 (2022)**

**https://doi.org/10.1186/s12866-021-02364-2**

Following the publication of the original paper [1], there’s an error on the first name of one of the authors. Mureil Derrien should be Muriel Derrien. Correct name is shown in the author group section above.

The original article has been corrected.
