# Supplementary figures and images for: Yoghurt consumption is associated with changes in the composition of the human gut microbiome and metabolome
Source: BMC Microbiol. 2022 Feb 3;22:39. doi: 10.1186/s12866-021-02364-2 (PMC8812230; doi:10.1186/s12866-021-02364-2)

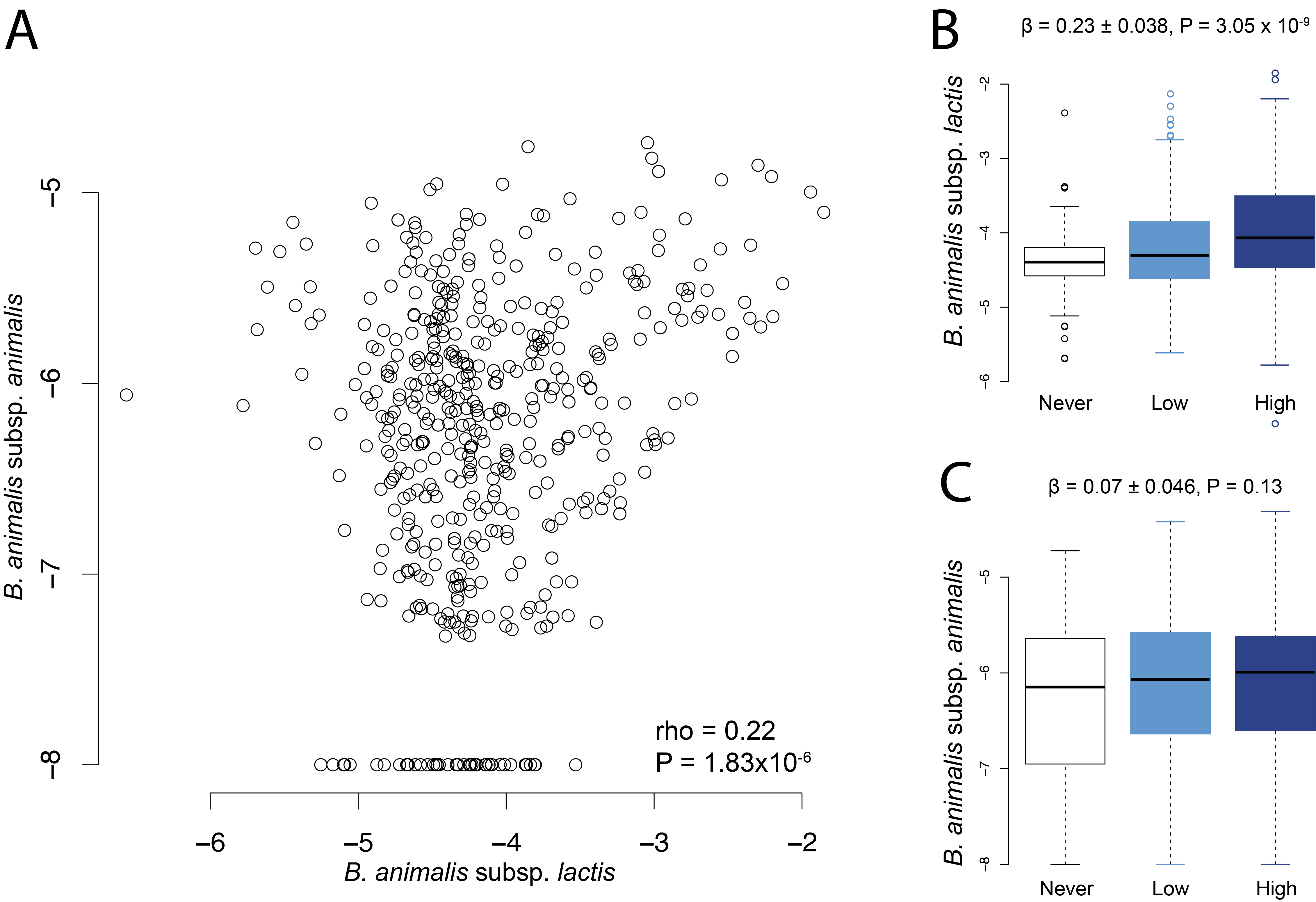

Supplement: Supplementary file 1 — Additional file 1: Supplementary Figure 1. Association between yoghurt consumption and B. animalis subsp. lactis . A. Scatter plot of the correlation between B. animalis subsp. lactis and B. animalis subsp. animalis. B. Boxplot picturing the association between frequency of yoghurt consumption and B. animalis subsp. lactis. C. Boxplot picturing the association between frequency of yoghurt consumption and B. animalis subsp. animalis. Results were obtained from linear regression (lme4 package in R) including family structure as random effect and age, BMI and sex as fixed effects. [file 12866_2021_2364_MOESM1_ESM.zip › Supp_Fig1.png]

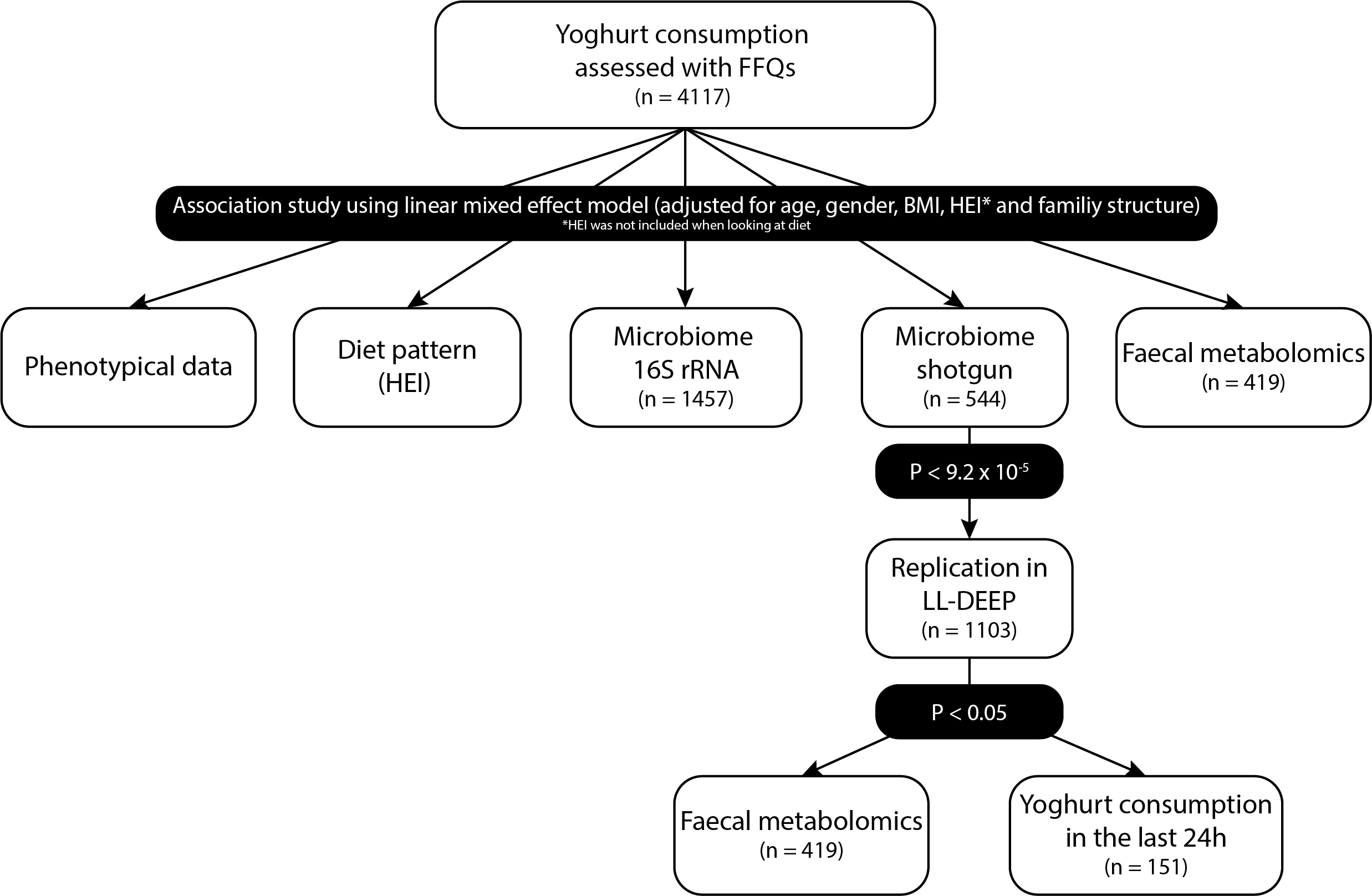

Supplement: Supplementary file 1 — Additional file 1: Supplementary Figure 1. Association between yoghurt consumption and B. animalis subsp. lactis . A. Scatter plot of the correlation between B. animalis subsp. lactis and B. animalis subsp. animalis. B. Boxplot picturing the association between frequency of yoghurt consumption and B. animalis subsp. lactis. C. Boxplot picturing the association between frequency of yoghurt consumption and B. animalis subsp. animalis. Results were obtained from linear regression (lme4 package in R) including family structure as random effect and age, BMI and sex as fixed effects. [file 12866_2021_2364_MOESM1_ESM.zip › Supplemnetary_Figure1.png]
